# Supplementary material for: A novel approach for modelling and classifying sit-to-stand kinematics using inertial sensors
Source: PLoS One. 2022 Oct 18;17(10):e0264126. doi: 10.1371/journal.pone.0264126 (PMC9578638; doi:10.1371/journal.pone.0264126)
Supplement: Attachment — Submitted filename: Response to reviewers-M Wairagkar.pdf [file pone.0264126.s009.pdf]

Response to Reviewers

Dear Editor and Reviewers,

We would like to thank the reviewers and editor for their comments and feedback on our paper "A novel approach for modelling and classifying sit-to-stand kinematics using inertial sensors" submitted to PLOS ONE. We accept and are grateful for all comments, which have resulted in a stronger revised manuscript.

A detailed response to all reviewer comments, organised and numbered by reviewer follows. Comments from reviewers are quoted directly in **bold**, followed with a complete response to each critique. For the convenience of reviewers, we have also quoted new text from manuscript directly relevant to each comment in *italics* as a part of our responses. All revisions are highlighted in the manuscript text in red.

<u>Reviewer #1 (Comments to authors):</u>

1) **In line 240, the authors mention they employed a single neuron ANN model, please clarify if a single hidden layer with single neuron is used. if it is so, please explicitly mention it in the manuscript.**

We thank the reviewer for their constructive comments and feedback. We have now clarified that the representation of our model could be generalised to artificial neural network with a single layer with a single neuron with sigmoid activation for regression to estimate thigh movement angular kinematics as follows in the manuscript in **line 238** as follows:

"To estimate stand-to-sit transition angle θ_T, we used an adaptable differentiable function with parameters that could be optimised to model the thigh motion kinematics. This function can be generalised and represented as an artificial neural network consisting of a single layer with a single neuron with sigmoid activation function in Eq 8 for regression (see Fig 4)."

2) **What is the learning algorithm used for the ANN model.**

We have estimated the angular kinematics of thigh using regression to fit sigmoid curve. The parameters of sigmoid model were estimated by least squares. We have now added the following description in **line 244**:

"In practice, to estimate thigh angular kinematics in this study, we performed regression analysis with sigmoid model described in Eq (8). Our approach can be generalised and represented in the form of a single neuron depicted by the model in Fig 4. The model parameters w and b determined the speed of transition and the centre of the sigmoid curve, the midpoint of the transition segment respectively. Input to the model t is the time window of transition segment. The value of w indicating the speed of transition was estimated, in the range of 0 and 1, by minimising the root mean squared error between the estimated angle and the ground truth reference angle of the thigh using least squares."

Reviewer #2 (Comments to authors):

The authors explore the employment of an unsupervised machine learning model in classifying sit-to-stand movements based on kinematics data captured and reconstructed using reduced inertial sensors. The paper is well written, the methodology employed is sound and the results were well discussed. The following are some minor comment(s):

We thank the reviewer for their encouraging feedback. We have addressed all comments as follows.

1) Line 192 - what linear classifier was used here?

We simply classified the stationary and transition states based on automatically identified threshold. We have now clarified it in the manuscript in **line 191** as follows:

"A threshold-based binary linear classification was performed to identify stationary state for feature values below the threshold and transition state for feature values above the threshold."

2) Line 257 - Was a single hidden layer with one hidden neuron used to estimate the thigh angle? You did mention that it was deemed sufficient, how was this conclusion made?

Yes, our model can be represented as a single layer ANN with only one neuron to estimate the thigh angle. We have now clarified it **in lines 238 and 262** as follows. This model was deemed sufficient for estimating simple sit-to-stand and stand-to-sit transitions based on the results described in Fig 6 comparing ground truth kinematics with estimated kinematics, Fig 7 Bland-Altman plots, and Table 1 root mean squared errors which show that the estimated output was accurate as compared to the ground truth motion capture data and the error between the two was low.

Line 238: *"To estimate stand-to-sit transition angle θ_T, we used an adaptable differentiable function with parameters that could be optimised to model the thigh motion kinematics. This function can be generalised and represented as an artificial neural network consisting of a single layer with a single neuron with sigmoid activation function in Eq 8 for regression (see Fig 4)."*

Line 262: *"This model analogous to a single layer with a single neuron was sufficient to estimate thigh angle (results are shown in the next section), however, it can be extended to a more complex artificial neural network with any bounded continuous differentiable activation function to perform regression to estimate the thigh angle."*

3) Also do report what optimisation algorithm used, e.g., Quasi-Newton, LM?

We have used least squares for regression to fit sigmoid curve which was used to estimate the angular kinematics of thigh. We have now added the following description to clarify this in **line 244**:

"In practice, to estimate thigh angular kinematics in this study, we performed regression analysis with sigmoid model described in Eq (8). Our approach can be generalised and represented in the form of a single neuron depicted by the model in Fig 4. The model

parameters w and b determined the speed of transition and the centre of the sigmoid curve, the midpoint of the transition segment respectively. Input to the model t is the time window of transition segment. The value of w indicating the speed of transition was estimated, in the range of 0 and 1, by minimising the root mean squared error between the estimated angle and the ground truth reference angle of the thigh using least squares."

Journal requirement comments:

1) **Please ensure that your manuscript meets PLOS ONE's style requirements, including those for file naming.**

We have checked our manuscript and ensured that it follows PLOS ONE's style requirements, including sections, section headings and figures.

2) **We note that Figure 5 includes an image of a [patient / participant / in the study]. Please amend the methods section and ethics statement of the manuscript to explicitly state that the patient/participant has provided consent for publication.**

We have received signed consent form from the individual in Figure 5 to publish this figure in the manuscript. We have clarified it in the manuscript by including the following in the ethics statement (**line 269**) and methods section (**line 307**): *"The individual seen in the figure in this manuscript has given written informed consent (as outlined in PLOS consent form) to publish the figure."*

3) **Please note that in order to use the direct billing option the corresponding author must be affiliated with the chosen institute.**

We have changed the corresponding author on our manuscript. The corresponding author is affiliated with the chosen institute for billing.

4) **Your ethics statement should only appear in the Methods section of your manuscript. If your ethics statement is written in any section besides the Methods, please delete it from any other section.**

We have now included a separate ethics statement in Methods section in **line 269**. Ethics statement is not included elsewhere.

We have addressed all reviewer comments and revised our manuscript based on the reviewers' feedback and journal style requirements. We thank the reviewers and the editor again and hope the revised manuscript meets their expectations.

Thank you,

Maitreyee

Maitreyee Wairagkar, PhD
Department of Mechanical Engineering
Imperial College London,
London SW7 1AL,
UK
